# Development of Coagulation Factor Probes for the Identification of Procoagulant Circulating Tumor Cells

**DOI:** 10.3389/fonc.2012.00110

**Published:** 2012-09-06

**Authors:** Garth W. Tormoen, Flor A. Cianchetti, Paul E. Bock, Owen J. T. McCarty

**Affiliations:** ^1^Department of Biomedical Engineering, Oregon Health and Science UniversityPortland, OR, USA; ^2^Department of Pathology, Microbiology and Immunology, Vanderbilt University School of MedicineNashville, TN, USA; ^3^Department of Cell and Developmental Biology, Oregon Health and Science UniversityPortland, OR, USA; ^4^Division of Hematology and Medical Oncology, Department of Medicine, Oregon Health and Science UniversityPortland, OR, USA

**Keywords:** tissue factor, coagulation, circulating tumor cell

## Abstract

Metastatic cancer is associated with a hypercoagulable state, and pathological venous thromboembolic disease is a significant source of morbidity and the second leading cause of death in patients with cancer. Here we aimed to develop a novel labeling strategy to detect and quantify procoagulant circulating tumor cells (CTCs) from patients with metastatic cancer. We hypothesize that the enumeration of procoagulant CTCs may be prognostic for the development of venous thrombosis in patients with cancer. Our approach is based on the observation that cancer cells are capable of initiating and facilitating cell-mediated coagulation *in vitro*, whereby activated coagulation factor complexes assemble upon cancer cell membrane surfaces. Binding of fluorescently labeled, active site-inhibited coagulation factors VIIa, Xa, and IIa to the metastatic breast cancer cell line, MDA-MB-231, non-metastatic colorectal cell line, SW480, or metastatic colorectal cell line, SW620, was characterized in a purified system, in anticoagulated blood and plasma, and in plasma under conditions of coagulation. We conclude that a CTC labeling strategy that utilizes coagulation factor-based fluorescent probes may provide a functional assessment of the procoagulant potential of CTCs, and that this strategy is amenable to current CTC detection platforms.

## Introduction

Cancer is a hypercoagulable state. Patients with cancer have a 4- to 10-fold increased risk of developing thrombosis, which is a significant source of morbidity and mortality for patients with cancer (Trousseau, 1865; Baron et al., 1998; Sorensen et al., 2000; Rickles and Levine, 2001; Falanga and Marchetti, 2009). Recurrent thrombosis can be clinically managed with anticoagulant therapy; however, the risk of bleeding complications associated with the use of anticoagulants has prevented routine prophylactic anticoagulation for patients with cancer who have not yet developed thrombosis (Akl et al., 2011). Therefore, a method to identify which cancer patients are at imminent risk to develop thrombosis would allow for an objective means by which to administer personalized anticoagulant prophylaxis, reducing the morbidity, and mortality for patients with cancer. There is currently a lack of laboratory assays capable of identifying which patients with cancer are at risk of developing thrombosis.

Blood coagulation is actuated by a system of serine proteases that are contained within the blood in their inactive, zymogen form. In health, activation of coagulation is restricted to sites of blood vessel injury through the localized exposure of tissue factor (TF), a transmembrane protein constitutively expressed by extravascular tissue not normally exposed to the circulating blood. As blood hemorrhages from an injured vessel, it comes into contact with TF-expressing cells outside of the vasculature. TF serves as the membrane receptor and protein cofactor of coagulation factor VIIa (FVIIa). The TF·FVIIa complex initiates the extrinsic blood coagulation pathway by activating factor X (FX) and factor IX (FIX). Activated factor X (FXa) and activated factor IX (FIXa) are initially inhibited by TF pathway inhibitor (TFPI) present in blood at a low concentration (∼2.4 nM) by forming a quaternary FXa·TF·FVIIa·TFPI complex (Baugh et al., 1998; Lu et al., 2004). FIXa forms the tenase complex with its protein cofactor FVIIIa on the surface of phosphatidylserine (PS)-containing cell membranes in the presence of calcium ion, which generates additional FXa. FXa produced on PS-containing cell membranes assembles, in a Ca^2+^-dependent manner, the prothrombinase complex with its protein cofactor factor Va (FVa). The prothrombinase complex converts prothrombin (FII) into thrombin (FIIa). FIIa cleaves fibrinogen into self-polymerizing, insoluble fibrin to form a plug at the injury site, effectively stemming blood loss. The localization of the procoagulant stimulus to the injury site, as well as anticoagulant effects of the endothelium downstream of the injury, serve to localize blood coagulation to the site of injury. However, pathologically excessive coagulation, or the initiation of coagulation at sites other than blood vessel injury, can result in thrombosis.

Hematogenous spread of metastatic cancer requires tumor cells to intravasate into blood vessels to navigate the bloodstream and establish distant metastases. The existence of tumor cells in the blood of patients with cancer has been known for over a century (Ashworth, 1869), yet only recently has technology allowed the routine cytological detection of these cells, hereafter referred to as circulating tumor cells (CTCs). CTCs have been demonstrated to be prognostic for overall patient survival, yet the impact of CTCs on cancer associated hypercoagulability has not been established (Cristofanilli et al., 2004; Danila et al., 2007; Cohen et al., 2008; de Bono et al., 2008). *In vitro*, cancer cell lines added to plasma are able to induce coagulation. The ability of cancer cell lines to clot plasma is abrogated by incubating with a TF-blocking antibody, or with Annexin V, which blocks the binding of coagulation factors to the PS-containing cancer cell membrane (Berny-Lang et al., 2011). Further, the clotting kinetics for plasma spiked with cancer cells is strongly dependent upon the number of cells added (Tormoen et al., 2011; Yates et al., 2011; Welsh et al., 2012). Therefore, it appears that cancer cells are wholly capable of cell-mediated coagulation *in vitro*, whereby they can initiate coagulation through surface expression of TF and facilitate the propagation of coagulation by binding and assembling coagulation factor complexes upon their cell membranes.

The ability for CTCs to facilitate coagulation in human disease has not been investigated, primarily due to the previously formidable technical challenge of identifying CTCs in the blood. Technological advancements have allowed the reliable detection of CTCs in patients with cancer through immunofluorescent labeling; specifically, cells that are cytokeratin positive, CD45 negative, and nucleated as apparent with DAPI staining are currently utilized to identify CTCs. On this basis, we sought to develop a functional probe that is amenable to fluorescence microscopic techniques to supplement CTC identification with the ability to characterize the procoagulant nature of CTCs. In this study, we characterized the binding of fluorescently labeled, active site-inhibited coagulation factors VIIa, Xa, and IIa to the metastatic breast cancer cell line, MDA-MB-231, non-metastatic colorectal cell line, SW480, or metastatic colorectal cell line, SW620, in a purified system and in blood plasma. Our approach is modeled after the presumed requirement for procoagulant cells to bind coagulation factors from plasma and subsequently assemble coagulation enzyme complexes on their surface to facilitate cell-mediated coagulation. We focused on coagulation factors in the TF-pathway of coagulation based upon the *in vitro* results demonstrating the TF- and phosphatidylserine (PS)-dependent pathways by which cancer cells mediate coagulation. We hypothesize that the identification and enumeration of procoagulant CTCs will be prognostic for venous thrombosis in patients with cancer.

## Materials and Methods

All reagents were purchased from Sigma or previously described sources (Berny-Lang et al., 2011). The function-blocking anti-factor XIa antibody 1A6 was obtained as previously described (Tucker et al., 2009). H-Gly-Pro-Arg-Pro-OH (GPRP) was purchased from Calbiochem.

### Fluorescent probes and reagents

Fluorescein isothiocyante (FITC)-conjugated TF monoclonal antibody was purchased from LifeSpan Bioscences. Human coagulation factors VIIa, Xa, IIa, and fluorescein-conjugated d-Phe-Pro-Arg-chloromethyl ketone (PPACK) were purchased from Haematologic Technologies (Essex Junction, VT, USA).

Coagulation factors were incubated with the fluorophore-conjugated PPACK as previously specified (Bock, 1992; Panizzi et al., 2006). In brief, active site inactivation was verified by comparing PPACK-bound coagulation factor activity toward the chromogenic substrates Spectrozyme FVIIa, Spectrozyme Xa, or Spectrozyme TH (American Diagnostica). Following inactivation, excess PPACK was removed by dialysis using a Slide-A-Lyzer^®^ MINI Dialysis Unit (Thermo Scientific) with 5 mM Hepes and 0.15 M NaCl (pH = 7.40).

### Cell culture

The metastatic breast cancer cell line, MDA-MB-231, non-metastatic colorectal cell line, SW480, and metastatic colorectal cell line, SW620, were obtained from American Type Cell Culture. Cells were cultured in Dulbecco’s Modified Eagle’s Medium (DMEM) containing 10% fetal bovine serum (Gibco) and maintained in a controlled environment at 37°C with 5% CO_2_/air atmosphere. Prior to each experiment, cells were detached from the culture flask by immersing in TrypLE Express (Gibco) for 20 min at 37°C, followed by resuspension in complete media, pelleted by subjecting to centrifugation at 210 × *g* for 5 min followed by final resuspension in serum-free DMEM. Resuspended cell concentrations were measured with a hemocytometer.

### Human blood and plasma

Blood samples were obtained and managed in accordance with Oregon Health and Science University Review Board approval. Human whole blood was collected from healthy volunteers by venipuncture into 1:9 v/v 3.2% sodium citrate. Platelet poor plasma (PPP) was obtained similarly, except that the collected blood was then subjected to centrifugation step at 2150 × *g* for 10 min, followed by removing the supernatant and mixing with the supernatant from two other donors. The pooled supernatant was then subjected to a second centrifugation step at 2150 × *g* for 10 min. The supernatant (PPP) was then removed, divided into 1 mL aliquots and stored at −80°C prior to use.

### Isolation of peripheral blood cells

To isolate human neutrophils, blood was collected 1:9 into 3.8% sodium citrate, followed by a 1:7 dilution into citrate-phosphate-dextrose as previously described (Itakura et al., 2011). In brief, 5 ml of blood suspension was layered over 5 ml of Polymorphprep and subjected to centrifugation at 500 × *g* for 45 min. The neutrophil band was extracted and diluted in Hank’s Balanced Salt Suspension (HBSS) to 50 ml, and subjected to centrifugation at 400 × *g* for 10 min. The supernatant was removed and the remaining cell pellet was resuspended in sterile water for 30 s, followed by diluting in 10 mL of 10X PIPES buffer (250 mM piperazine-*N*,*N*′*bis* [2-ethanesulfonic acid], 1.1 mM NaCl, 50 mM KCl, pH = 7.40), then the volume increased to 50 mL with HBSS, and subjected to a final centrifugation step at 400 × *g* for 10 min. Cells were counted with a hemocytometer and diluted to a final concentration of 10^6^/mL.

To isolate human platelets, blood was collected as above but then subjected to centrifugation at 200 × *g* for 20 min as previously described (White-Adams et al., 2009). In brief, the supernatant containing plasma and platelets was incubated with 0.10 μg/mL of prostacyclin and subjected to centrifugation at 1000 × *g* for 10 min. The platelet pellet was resuspended in modified Tyrode’s buffer (129 mM NaCl, 0.34 mM Na_2_HPO_4_, 2.9 mM KCl, 12 mM NaHCO_3_, 20 mM HEPES, 5 mM glucose, 1 mM MgCl_2_; pH = 7.30).

### Clotting times

MDA-MB-231, SW480 or SW620 cells were diluted from 3 × 10^6^ to 1.5 × 10^3^ cells/ml in serum-free DMEM. Next, 50 μL of cell suspension or vehicle (DMEM) was mixed with 50 μL of PPP for 180 s at 37°C. Then, 50 μL of 25 mM CaCl_2_ was added and the time required for the plasma to clot was measured on a KC4 coagulation analyzer (Trinity Biotech, Bray, Co., Wicklow, Ireland). To determine the mechanism of the cancer cell procoagulant activity, 50 μL of 3 × 10^5^ cells/mL were pretreated with a function-blocking anti-TF mAb (50 μg/mL) or the phosphatidylserine function-blocking ligand Annexin V (20 μg/mL) for 5 min at room temperature prior to mixing with plasma. Further, PPP was pretreated with the FXa inhibitor Rivaroxaban (20 μg/mL) for 5 min prior to mixing with cells.

Experiments were performed in duplicate and the average clotting time reported. Clotting time experiments were repeated 3–9 times and plotted as mean ± the standard error of the mean. Statistically significant differences were evaluated using a Student’s *T* tests with Bonferroni’s correction against untreated cells (
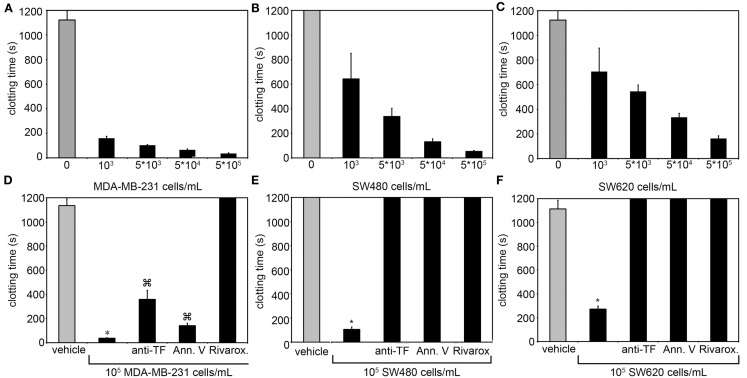
 for *p*-value <0.05) or vehicle (* for *p*-value <0.05).

### Flow cytometry

One-hundred thousand MDA-MB-231 or SW620 cells were suspended in 50 μl of PBS, PPP, or PPP treated with the anti-FXI antibody 1A6 (12.5 μg/mL) and the fibrin polymerization inhibitor Gly-Pro-Arg-Pro-OH (GPRP 10 mM), and 8.3 mM CaCl_2_ (final concentration). Cell suspensions were incubated with fluorescently labeled coagulation factors FVIIa (50–500 nM), FXa (100–5000 nM) or FIIa (100-10000 nM), or FITC-conjugated anti-TF (10–100 μg/mL) for 30 min at room temperature. Then labeled cells were diluted to 500 μL with PBS and characterized using a FACS Calibur flow cytometer with CellQuest Pro acquisition and analysis software (Becton Dickinson, Franklin Lakes, NJ, USA) as previously described (McCarty et al., 2002).

### Static adhesion

Coverslips (#1.5 12 mm; Fischer Scientific) were etched for 30 s in a 70% nitric acid bath, immersed in deionized H_2_0 (*R* = 18.2 MΩ.cm), rinsed in ethanol, and allowed to air dry. Etched and dried coverslips were then placed in individual wells of a 24-well plate. Five hundred μL of 4% 3-aminopropyltriexothysilane in ethanol was added to the wells and allowed to coat for 2 min. Coverslips were then washed once in ethanol and submerged in H_2_0 prior to performing the experiments.

Three hundred thousand MDA-MB-231, SW480, or SW620 cells in serum-free DMEM were dispensed onto the etched coverslips and allowed to adhere for 60 min at 37°C. Non-adherent cells were removed by washing with PBS. Recalcified plasma containing the anti-FXIa antibody 1A6 (12.5 μg/mL) and 10 mM GPRP was dispensed over immobilized cells and allowed to incubate for 30 min at room temperature. Cells were washed in PBS, and incubated with fluorescently labeled coagulation factors FVIIa (500 nM), FXa (5 μM), or FIIa (10 μM) or FITC-conjugated anti-TF mAB (50 μg/mL) in PBS for 30 min at room temperature. Labeled cells were washed in PBS, fixed with 3.7% paraformaldehyde, washed in triplicate, and mounted in Fluoromount G (Southern Biotech) and kept at 4°C overnight. Labeled, adherent cells were imaged on a Zeiss Axiovert 200 M at 40× with a Zeiss EC Plan-Neofluar 0.75 NA objective using fluorescence and differential interference contrast (DIC) microscopy. A minimum of three images were recorded from each experimental condition, with representative images shown for each condition.

Binding of coagulation factors to purified populations of peripheral blood cells was performed by dispensing 300 μL of cells onto silanized coverslips and allowing them to adhere for 60 min at 37°C. Cells were then washed and incubated with PBS (vehicle) or PBS containing active site-inhibited, fluorescent coagulation factor probes for 30 min at room temperature. Cells were then washed in PBS, fixed, and mounted as described above.

## Results

### Clotting times

**Figure 1:**
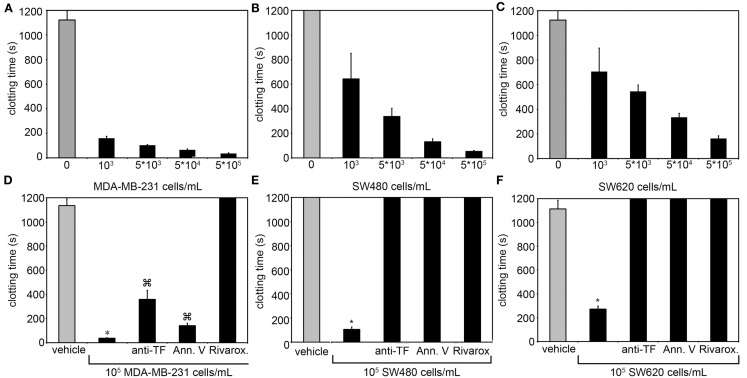
**Clotting times for human plasma containing MDA-MB-231, SW480, or SW620 cells**. The addition of MDA-MB-231, SW480, and SW620 cells shortened clotting times of plasma in a cell count dependent manner. **(A–C)** The ability for these cells to coagulate plasma was inhibited with a function-blocking antibody to tissue factor (TF) or Annexin V, and completely abrogated with a Factor Xa inhibitor. **(D–F)** Human sodium citrate anticoagulated plasma was pretreated with vehicle or the FXa inhibitor rivaroxaban for 5 min at room temperature. MDA-MB-231, SW480, or SW620 cells were pretreated with vehicle, a neutralizing antibody to TF (anti-TF, 50 μg/mL) or the phosphatidylserine binding protein Annexin V (20 μg/mL) for 5 min at room temperature. Cells and plasma were then mixed together for 3 min on a KC4 coagulation analyzer prior to recalcification to 8.3 mM (final concentration). Clotting time experiments were performed in duplicate for each condition and reported as the average. Each experiment was independently repeated 3–9 times. Data are reported as mean ± SEM. *P < 0.05 vs. the absence of cells. 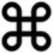 *P* < 0.05 vs. vehicle pretreated cells.

To investigate whether the metastatic breast cancer cell line, MDA-MB-231, non-metastatic colorectal cell line, SW480, or metastatic colorectal cell line, SW620, were sufficient to initiate and propagate blood coagulation, washed cells were suspended in serum-free DMEM and added to recalcified human plasma. The subsequent time required for the plasma to clot (i.e., clotting times) was measured as a function of cell count in a coagulometer (Figures 1A–C). All three cancer cell lines hastened the time for plasma to clot as compared to vehicle (DMEM) and the clotting times depended upon the number of cancer cells added to the plasma. To determine the role for cancer cell-expressed TF in initiating clotting, a function-blocking anti-TF mAb (50 μg/mL) was added to the cancer cells prior to mixing with plasma. Our results show that the anti-TF mAb abrogated the ability for SW480 and SW620 cells to clot plasma, and prolonged the clotting times for MDA-MB-231 cells (288.6 s vs. 38.5 s, respectively). We next designed experiments to determine whether cancer cell surface exposed acidic phospholipids were required for clotting. Cancer cells were pretreated with Annexin V (20 μg/mL), which binds to and functionally blocks the ability for PS to bind clotting factors and assemble enzyme complexes on a cell surface. Pretreating SW480 and SW620 cells with Annexin V abrogated the ability of these cells to clot plasma. Pretreatment of MDA-MB-231 cells with Annexin V prolonged clotting times in a concentration-dependent manner (131.2 s at 20 μg/mL vs. 229.1 s at 40 μg/mL). Finally, pretreating the plasma with the FXa inhibitor, Rivaroxaban, prior to mixing with the cancer cells completely abrogated the ability of the three cancer cell lines tested to clot plasma. Taken together, our data demonstrate that MDA-MB-231, SW480, and SW620 cells are procoagulant in a TF, PS, FXa, and cell count dependent manner (Figures 1D–F).

### Flow cytometry of labeled cells

#### Labeling of cancer cells in a purified system

**Figure 2:**
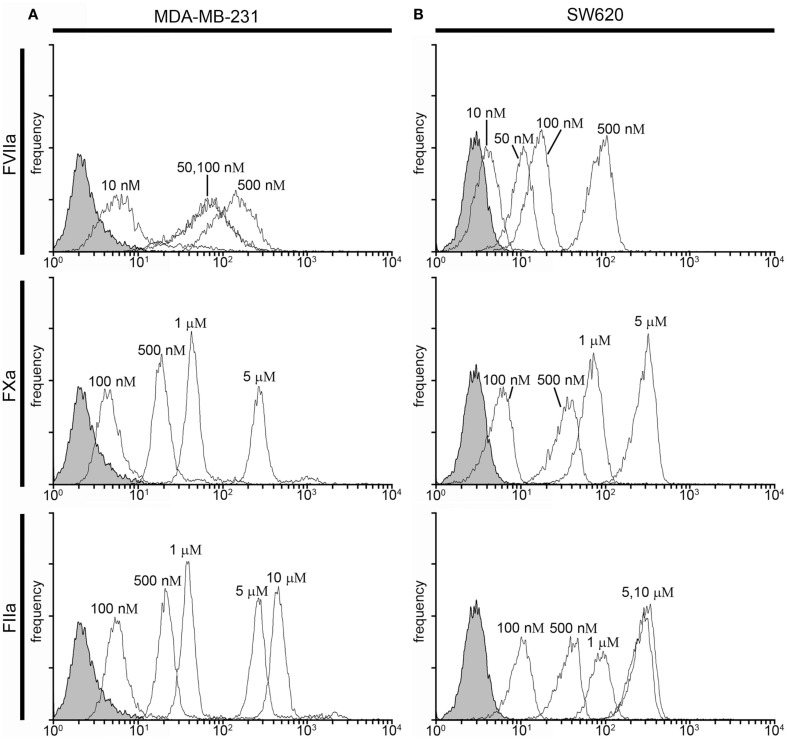
**Characterization of fluorescent coagulation factor probe binding to MDA-MB-231 and SW620 cells suspended in DMEM**. One-hundred thousand MDA-MB-231 **(A)** or SW620 cells **(B)** were incubated with vehicle or fluorescently modified, active site-inhibited coagulation factors FVIIa (10–500 nM), FXa (100–5000 nM), or FIIa (100–10,000 nM) for 30 min at room temperature. Cells were then diluted 10-fold in sterile-filtered phosphate-buffered saline (PBS, pH = 7.40) and analyzed by flow cytometry. Shaded histograms represent background fluorescence while white histograms represent labeled cells at the fluorescent probe concentrations shown.

We next investigated whether fluorescently labeled, active site-inhibited coagulation factors could be used to label procoagulant cancer cells in a purified system. For this, we utilized the MDA-MB-231 and SW620 cancer cell lines, as they were shown to have the highest and lowest procoagulant activities of the cancer cell lines we tested, respectively, and we aimed to determine if coagulation factor-based probes could label both cell lines as well as contrast the labeling efficacy between cell lines. MDA-MB-231 and SW620 cells were suspended in serum-free DMEM and incubated with either vehicle or active site-blocked, fluorescently labeled FVIIa (50 nM), FXa (0.5–5 μM), or FIIa (0.5–10 μM) for 30 min at room temperature. Samples were diluted 10-fold in phosphate-buffered saline (PBS) and fluorescence recorded with flow cytometry. Figure 2 shows the fluorescence intensity histogram for labeled cells vs. vehicle treated controls. The surface expression of TF was verified by staining of the cell lines with a FITC-conjugated anti-TF mAb (data not shown).

Factor VIIa-labeling of MDA-MB-231 cells was clearly evident at 50 nM [mean fluorescence intensity (MFI) = 67 vs. 2.6 for unstained cells] and did not change at 100 nM (MFI = 72; Figure 2A). Factor VIIa-labeling of SW620 cells was evident at 50 nM (MFI = 10 vs. 2.86 for unlabeled cells), with further MFI increases observed at 100 and 500 nM (MFI = 16 and 88, respectively; Figure 2B). FXa-labeling of MDA-MB-231 and SW620 cells was evident at 500 nM (MFI = 21.05 for MDA-MB-231 and MFI = 33.31 for SW620), with further increases in fluorescence intensity for labeling at 1 and 5 μM (MFI = 49 and 324 for MDA-MB-231, and MFI = 67 and 281 for SW620, respectively). FIIa (thrombin) labeling of MDA-MB-231 cells was not apparent at 100 nM (MFI = 6.17) while SW620 cells (MFI = 9.91) were labeled at 100 nM. MDA-MB-231 cells showed increases in labeling intensity with FIIa-based probes from 500 nM to 10 μM (MFI = 24, 42, 305, and 531 for 500 nM, 1, 5, and 10 μM, respectively). SW620 cells showed increases in labeling intensity from 500 nM to 1 μM (MFI = 37, 90, 282 for 500 nM, 1 and 5 μM, respectively), with no further increase seen at 10 μM (MFI = 258). Labeling of cancer cells in a purified system showed cell and factor-specific characteristics for labeling efficacy. Our data show that a concentration of 50 nM FVIIa-based probe was sufficient to label both the MDA-MB-231 and SW620 cells, while a concentration of 500 nM of the FXa- or FIIa-based probes was required to label both MDA-MB-231 and SW620 cells.

#### Labeling of cancer cells in human plasma

**Figure 3:**
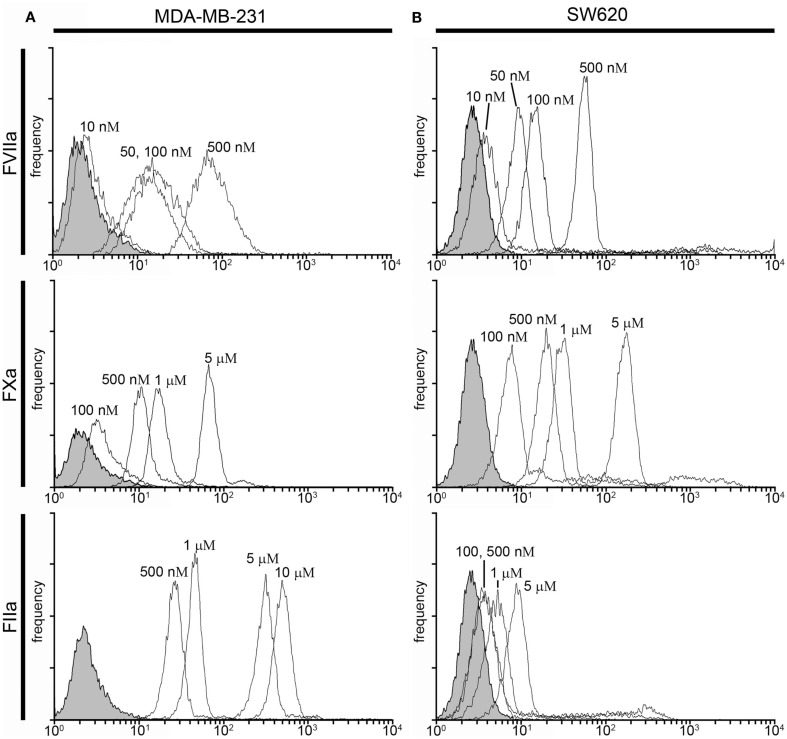
**Characterization of fluorescent coagulation factor probe binding to MDA-MB-231 and SW620 cells in human plasma**. One-hundred thousand MDA-MB-231 **(A)** or SW620 cells **(B)** were incubated with vehicle or fluorescently modified, active site-inhibited coagulation factors FVIIa (10–500 nM), FXa (100–5000 nM), or FIIa (100–10,000 nM) in 50 μL of PPP for 30 min at room temperature. Cells were then diluted 10-fold in sterile-filtered phosphate-buffered saline (PBS, pH = 7.40) and analyzed by flow cytometry. Shaded histograms represent background fluorescence while white histograms represent labeled cells at the fluorescent probe concentrations shown.

We next investigated whether fluorescently labeled coagulation factors could be used to label procoagulant cancer cells in plasma. For this, MDA-MB-231and SW620 cells were suspended in sodium citrate anticoagulated PPP containing vehicle or fluorescently labeled FVIIa (50–500 nM), FXa (0.5–5 μM), or FIIa (0.5–10 μM) for 30 min at room temperature. Samples were diluted 10-fold in PBS and fluorescence measured with flow cytometry. Figure 3 shows the fluorescence intensity histogram for labeled cells vs. vehicle treated controls. The surface expression of TF was verified by staining of the cell lines with a FITC-conjugated anti-TF mAb (data not shown).

Factor VIIa-labeling of MDA-MB-231 cells in plasma was evident at 50 nM (MFI = 13.8 vs. 2.4 for unlabeled cells) and fluorescence labeling increased at probe concentrations of 100 nM (MFI = 18 and 81 for 100 and 500 nM, respectively; Figure 3A). FVIIa-based probe labeling of SW620 cells was evident at 50 nM (MFI = 11.7 vs. 2.8 for unlabeled cells) in plasma with a further increases in fluorescence intensity at higher probe concentrations (MFI = 20 and 76 at 100 and 500 nM, respectively; Figure 3B). Labeling of MDA-MB-231 cells with the FXa-based probe in plasma was not evident at 100 nM (MFI = 3.9 vs. 2.4 for unlabeled cells), but could be seen at 500 nM (MFI = 11.3), with further increases in fluorescence labeling at higher probe concentrations (MFI = 18.9 and 74.2 for 1 and 5 μM, respectively). Labeling of SW620 cells with the FXa-based probe in plasma was observed at 100 nM (MFI = 8.05 vs. 2.8 for unlabeled cells) and increases in fluorescence intensity were observed at higher probe concentrations (MFI = 22, 35, and 210 at 500 nM, 1 and 5 μM, respectively). FIIa-labeling of MDA-MB-231 cells was observed at 500 nM (MFI = 27 vs. 2.6 for unlabeled cells) with further increases in fluorescence intensity seen with probe concentration (MFI = 47, 321, and 515 for 1, 5, and 10 μM, respectively). In contrast, the FIIa-probe did not label SW620 cells at or below 1 μM FIIa-probe concentration (MFI = 3.8, 4.2, and 6.8 for 100, 500, and 1000 nM, respectively).

#### Labeling of cancer cells in plasma under conditions of coagulation

**Figure 4:**
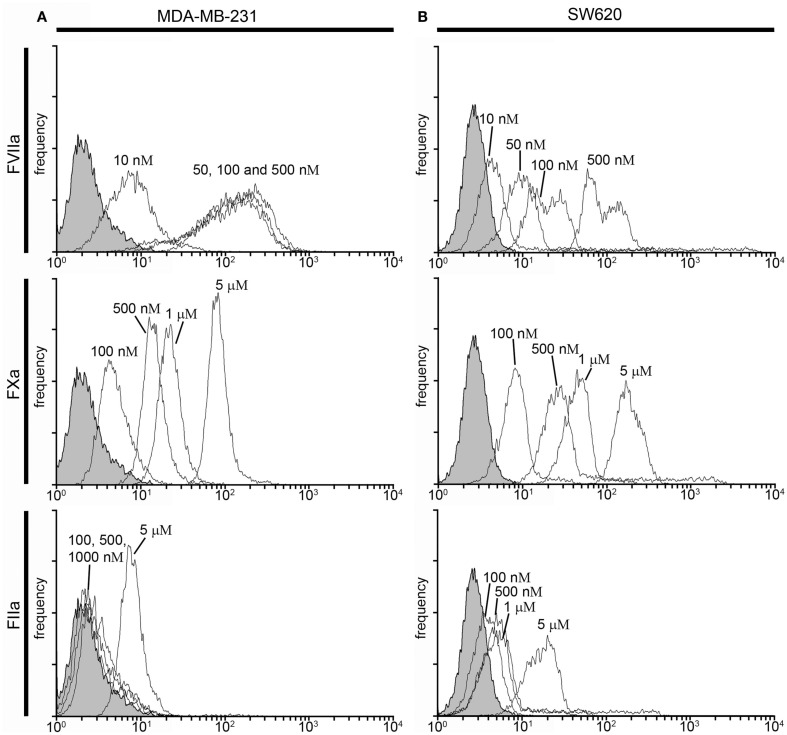
**Characterization of fluorescent coagulation factor probe binding to MDA-MB-231 and SW620 cells in human plasma under conditions of coagulation**. One-hundred thousand MDA-MB-231 **(A)** or SW620 cells **(B)** were incubated with vehicle or fluorescently modified, active site-inhibited coagulation factors FVIIa (10–500 nM), FXa (100–5000 nM), or FIIa (100–10,000 nM) in 50 μL of recalcified PPP (8 mM, final Ca^2+^ concentration) containing the fibrin polymerization blocker GPRP (10 mM) and the FXIa-blocking antibody 1A6 (12.5 μg/mL) for 30 min at room temperature. Cells were then diluted 10-fold in sterile-filtered phosphate-buffered saline (PBS, pH = 7.40) and analyzed by flow cytometry. Shaded histograms represent background fluorescence while white histograms represent labeled cells at the fluorescent probe concentrations shown.

In the presence of a procoagulant stimulus, such as a procoagulant cancer cell, coagulation factors in plasma undergo limited proteolysis to become activated. In addition, the presence of Ca^2+^ ions may present Ca-dependent binding sites on cells which are inaccessible in the presence of the anticoagulant, sodium citrate. To determine if coagulation factor-based probes could be used to label procoagulant cancer cells under conditions of coagulation, cancer cells were suspended in PPP pretreated with an anti-FXIa antibody and GPRP and recalcified to 8 mM Ca^2+^ containing vehicle or fluorescently labeled FVIIa (50–500 nM), FXa (0.5–5 μM), or FIIa (0.5–10 μM) for 30 min at room temperature. Figure 4 shows the fluorescence intensity histogram for labeled cells vs. vehicle treated controls.

FVIIa-labeling of both cell types was evident at 50 nM (MDA-MB-231 MFI = 102 vs. 2.4 for unlabeled cells (Figure 4A), SW620 MFI = 11.4 vs. 2.8 for unlabeled cells (Figure 4B), and increases in FVIIa-based probe concentrations showed minimal effect on MDA-MB-231 fluorescence intensity (MFI = 122 and 159 for 100 and 500 nM, respectively). SW620 cells exhibited increases in fluorescence intensity with FVIIa-based probe concentrations of 100 and 500 nM (MFI = 23.6 and 112.3, respectively). FXa-labeling of MDA-MB-231 cells was seen at 500 nM (MFI = 15.5 vs. 2.4 for unlabeled cells), and the fluorescence intensity increased with FXa-based probe concentration (MFI = 24 and 90 for 1 and 5 μM, respectively). FXa-based probe labeling of SW620 cells was seen at 500 nM (MFI = 27.3 vs. 2.8 for unlabeled cells) and further increases in fluorescence intensity were seen at FXa-based probe concentrations of 1 and 5 μM, respectively. The FIIa-based probe labeled SW620 cells at but not below 5 μM (MFI = 21 vs. 2.8 for unlabeled cells), while MDA-MB-231 cells were not clearly labeled at 5 μM under conditions of coagulation (MFI = 8.4 vs. 2.4 for unlabeled cells).

### Imaging of immobilized cells

#### Labeling of immobilized cancer cells in a purified system

**Figure 5:**
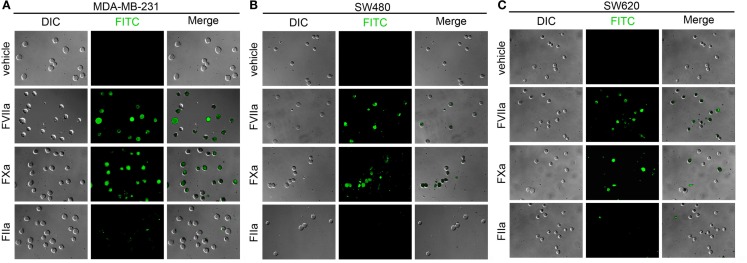
**Characterization of fluorescent coagulation factor probe binding to immobilized MDA-MB-231, SW480, and SW620 cells in DMEM**. MDA-MB-231 **(A)**, SW480 **(B)**, and SW620 cells **(C)** were immobilized on silanized glass slides prior to incubation with DMEM (vehicle) or DMEM containing fluorescently labeled coagulation factors FVIIa (500 nM), FXa (5 μM), or FIIa (10 μM) for 30 min at room temperature, washed in DMEM and fixed in 3.7% paraformaldehyde. Immobilized cells were imaged using differential interference contrast (DIC) and fluorescence microscopy.

Due to the extreme rarity with which CTCs are present in the blood of patients with cancer, flow cytometry is not routinely utilized to detect CTCs. Rather, various plating or lab-on-chip methods are utilized in combination with fluorescent labels to identify and/or isolate CTCs from a population of cells that consists of both normal blood cell constituents and CTCs (Nagrath et al., 2007; Gleghorn et al., 2010; Marrinucci et al., 2012). We designed a series of experiments to determine whether our labeling strategy was amenable to a cell processing protocol that utilizes cancer cells being plated onto glass slides. We immobilized MDA-MB-231, SW480, and SW620 cells on functionalized glass surfaces and exposed them to fluorescently labeled FVIIa (500 nM), FXa (5 μM), FIIa (10 μM). DIC, fluorescence, and merged images are shown in Figure 5 for MDA-MB-231, SW480, and SW620 cells. The images showed that the MDA-MB-231 cells were robustly labeled with the FVIIa and FXa probes. The FVIIa and FXa probes weakly labeled the SW480 cells and SW620 cells. The FIIa-probe failed to label any of the three cell lines.

#### Labeling of immobilized cancer cells following exposure to plasma under conditions of coagulation

**Figure 6:**
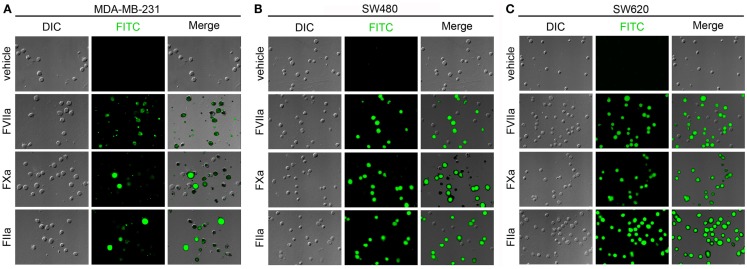
**Characterization of fluorescent coagulation factor probe binding to immobilized MDA-MB-231, SW480, and SW620 cells in the presence of coagulation**. MDA-MB-231 **(A)**, SW480 **(B)**, and SW620 cells **(C)** were immobilized on silanized glass slides and incubated with recalcified human plasma containing the anti-FXIa antibody, 1A6, and the fibrin polymerization inhibitor, GPRP, for 30 min at room temperature. Cells were then washed and treated with PBS (vehicle) or PBS containing fluorescently labeled coagulation factors FVIIa (500 nM), FXa (5 μM), or FIIa (10 μM) for 30 min at room temperature, washed in PBS, and fixed in 3.7% paraformaldehyde. Immobilized cells were imaged using differential interference contrast (DIC) and fluorescence microscopy.

Our next set of experiments were designed to determine whether fluorescently labeled coagulation factor probes could label cells that had been exposed to blood plasma under conditions of coagulation. We immobilized MDA-MB-231, SW480, and SW620 cells on functionalized glass surfaces, exposed the immobilized cells to recalcified plasma, and then incubated the slides with fluorescently labeled FVIIa (500 nM), FXa (5 μM), or FIIa (10 μM). DIC, fluorescence, and merged images are shown in Figure 6 for MDA-MB-231, SW480, and SW620 cells. The images showed that all coagulation factor-based probes labeled at least a portion of the adherent cancer cells for all three cancer cell lines. The FVIIa probe labeled all the adherent MDA-MB-231 cells. Heterogeneous FVIIa-labeling was observed for both the SW480 and SW620 cell lines, with some of the adherent cells labeling brightly, while other cells on the same slide were not labeled by the FVIIa probe. The FXa probe showed complete labeling of all three cell lines, but pronounced heterogeneity in labeling was noted as some cells were brightly labeled and others showed dim labeling by the FXa probe. The FIIa-probe showed complete labeling of the MDA-MB-231 and SW620 cell lines and heterogeneous labeling of the SW480 cells.

#### Labeling of immobilized cancer cells in whole blood

**Figure 7:**
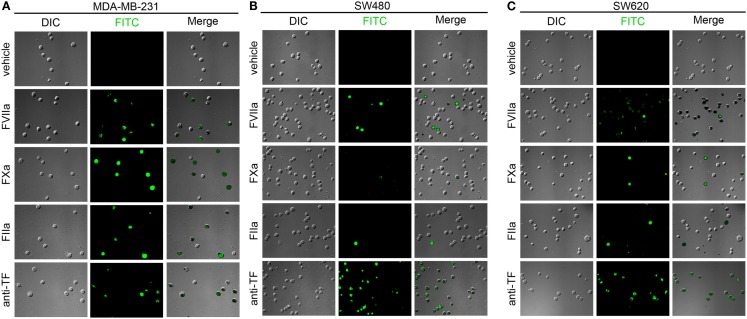
**Characterization of fluorescent coagulation factor probe binding to immobilized MDA-MB-231, SW480, and SW620 cells in whole blood**. MDA-MB-231 **(A)**, SW480 **(B)**, and SW620 cells **(C)** were immobilized on silanized glass slides and incubated with sodium citrate anticoagulated whole blood (vehicle) containing fluorescently labeled coagulation factors FVIIa (500 nM), FXa (5 μM), FIIa (10 μM), or FITC-conjugated anti-TF mAb (50 μg/mL) for 30 min at room temperature, washed in PBS and fixed in 3.7% paraformaldehyde. Immobilized cells were imaged using differential interference contrast (DIC) and fluorescence microscopy.

Following our experiments in cell-free labeling buffers, we next determined whether coagulation factor-based probes could be used to label cancer cells in whole blood. Immobilized MDA-MB-231, SW480, and SW620 cells were incubated with anticoagulated whole blood containing either vehicle, or fluorescent, active site-inhibited FVIIa (500 nM), FXa (5 μM), FIIa (10 μM), or FITC-conjugated anti-TF mAb (50 μg/mL) for 30 min at room temperature. DIC, fluorescence, and merged images are shown in Figure 7. The FVIIa probe showed heterogeneous labeling of the MDA-MB-231 cells, SW480, and SW620 cells, although the labeling of the SW480 cells was greatly diminished as compared to cells which had been exposed to plasma under conditions of coagulation (Figure 6). Heterogeneous labeling of all three cell lines with the FXa and FIIa-probes was observed, with very few SW480 or SW620 cells labeled. All three cell lines were labeled with the anti-TF mAb in whole blood.

#### Labeling of immobilized platelets and neutrophils

**Figure 8:**
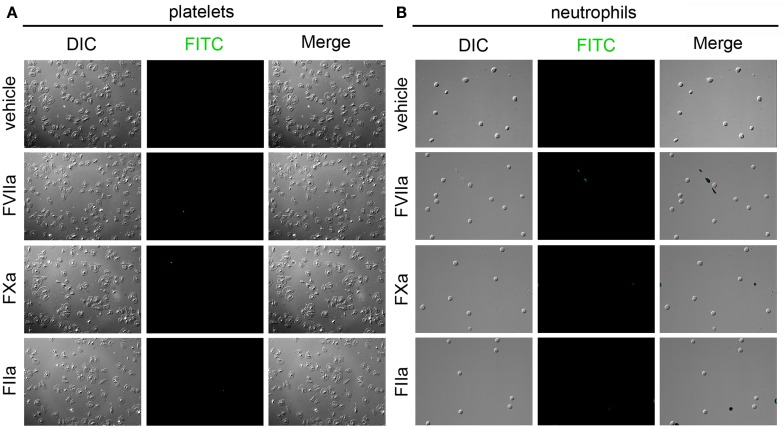
**Characterization of fluorescent coagulation factor probe binding to immobilized human neutrophils and platelets**. Human platelets **(A)** and neutrophils **(B)** were immobilized on silanized glass slides and incubated with PBS (vehicle) containing fluorescently labeled coagulation factors FVIIa (500 nM), FXa (5 μM), or FIIa (10 μM) for 30 min at room temperature, washed in PBS and fixed in 3.7% paraformaldehyde. Immobilized cells were imaged using differential interference contrast (DIC) and fluorescence microscopy.

Following the reduced labeling of the MDA-MB-231 cells, SW480, and SW620 cells in whole blood as compared to cell-free labeling solutions, we designed a set of experiments to determine if peripheral blood cells might be binding the probe in solution, and thereby causing diminished labeling of immobilized cancer cells. Neutrophils and platelets were purified from peripheral blood draws and immobilized onto silanized glass slides, and incubated with fluorescent FVIIa (500 nM), FXa (5 μM), and FIIa (10 μM). Coagulation factor-based probes failed to bind immobilized peripheral blood cells in a purified system (Figure 8). Our data show that neither the FVIIa (500 nM), FXa (5 μM), FIIa (10 μM) probes labeled purified human platelets (Figure 8A) or neutrophils (Figure 8B). In a complementary experiment, addition of purified human neutrophils to plasma failed to reduce clotting times (605.3 s vs. 672.7 s for vehicle and 10^5^/mL neutrophils, respectively) demonstrating that purified human neutrophils did not exhibit a procoagulant phenotype. Similar fluorescent coagulation factor labeling results were observed in whole blood (data not shown).

## Discussion

Metastatic disease accounts for the majority of cancer deaths. VTE events are significant contributors to metastatic disease, with reports indicating 18% of cancer deaths resulting from VTE. For instance, lung adenocarcinoma patients have an estimated 20-fold higher risk for developing VTE than the general population; however, not all patients develop VTE, making the bleeding risks of anticoagulating the entire adenocarcinoma patient population unacceptable. No current technology is available to objectively predict patient risk for VTE in order to personalize anticoagulant prophylaxis therapy. CTC counts have not been evaluated as a potential biomarker for risk to develop cancer associated thrombosis.

In this study, we demonstrate the use of fluorescently modified, active site-inhibited coagulation factors to label procoagulant cancer cells. The metastatic breast cancer cell line, MDA-MB-231, and metastatic colorectal cell line, SW620, were used due to the fact that these cell lines possess the ability to survive circulation in the blood to establish hematogenous metastases in murine models of cancer metastasis (Zhang et al., 1991; Sampson-Johannes et al., 1996). The non-metastatic colorectal cell line, SW480, was derived from the primary tumor from the same individual from which the SW620 was derived. We determined that all three cell lines exhibited a procoagulant phenotype, with the MDA-MB-231 cells resulting in the highest procoagulant activity, while the SW620 cells had the lowest procoagulant activity. The procoagulant activity of the MDA-MB-231, SW480, and SW620 cell lines could be reduced or abrogated by a function-blocking antibody to TF, or by pretreatment with Annexin V. Annexin V blocks the binding of coagulation factors that contain γ-carboxyglutamic acid domains (Gla domains) to PS on the cell membrane. These results support the notion that TF and PS exposure is a general phenomenon seen for cancer cell-mediated coagulation *in vitro*, and support a role for CTC-mediated coagulation as a potential contributor to the hypercoagulability seen for patients with cancer.

Previous studies have shown that cancer cell procoagulant activity correlates better with PS exposure than with overall TF expression levels, supporting a role for cell membrane effects in regulating procoagulant activity as opposed to surface TF expression (Barrowcliffe et al., 2002; Pickering et al., 2004). Therefore, we aim to develop a function-based CTC labeling strategy to determine whether CTCs are procoagulant, and whether CTC enumeration and procoagulant characterization strategies are clinically useful in predicting thrombosis in patients with cancer. We hypothesized that coagulation factors themselves would serve as specific, functional probes with which to identify procoagulant cells. Our approach was based upon the requirement for coagulation factors to for surface-assembled enzyme complexes on the surfaces of procoagulant cells in order to catalyze intermediate steps of the coagulation cascade. We focused on the coagulation factors FVIIa, FXa, and FIIa, as they are key components of the extrinsic (TF) pathway of coagulation.

Our results with flow cytometry show that the cancer cell lines MDA-MB-231, SW480, and SW620 bind coagulation factor probes. Immobilized cells show that cancer cells exhibited heterogeneity in their ability to bind various fluorescently modified active site-inhibited coagulation factors. For instance, we observed heterogeneous labeling of SW480 with the FVIIa probe in a both a purified system and in whole blood. Binding heterogeneity was observed over a range of probe concentrations (data not shown), suggesting the heterogeneity was not due to a scarcity of probe concentration. Further, the *K*_D_ for FVIIa to TF is in the pM range, five orders of magnitude below our labeling concentration. A stronger binding of FVIIa could suggest that these cells are more procoagulant than cells that show weak binding, however, currently no method can be used to determine individual cell procoagulant activity. Moreover, protease activated receptor 2 (PAR2) is another known receptor for FVIIa besides TF. MDA-MB-231 and SW620 cells are known to express PAR2, while SW480 cells have been shown to have little PAR2 surface expression (Morris et al., 2006; Zhou et al., 2008). Whether coagulation factor probe binding levels correlate with procoagulant activity or whether PAR2 expression levels affect binding of FVIIa probe is a focus of future studies.

FIIa-based probes brightly labeled immobilized MDA-MB-231, SW480, and SW620 cells in the presence of coagulation. In contrast, FIIa-probes only weakly labeled these cell lines in whole blood and failed to label any cells in DMEM. One prominent difference between FIIa and the other coagulation factor probes is the absence of the Gla domain for FIIa. The Gla domain mediates calcium ion-dependent binding of vitamin K-dependent coagulation factors to PS-containing procoagulant cell membranes. Calcium-dependent binding may account for differences in the FVIIa or FXa probe labeling as compared to FIIa in DMEM, which contains calcium, but fails to account for differences in whole blood in the presence of the calcium-chelator sodium citrate. As FIIa binds fibrinogen and fibrin, it is possible that FIIa labeled cancer cells that are coated in fibrin, a phenomenon that would be expected after exposing a procoagulant cancer cell to plasma under conditions of coagulation. However, using flow cytometry, we observed labeling of cancer cells with a FIIa-based probe in purified systems, suggesting an alternate mechanism for binding of FIIa to the cancer cell surface. Our future work will be focused on identifying the mechanism(s) of FIIa-cancer cell binding.

In this study, we demonstrated the use of fluorescently modified, active site-inhibited coagulation factors to label procoagulant cancer cells. We demonstrated that coagulation factors based probes bound to cancer cell lines in purified systems and in whole blood, yet failed to bind to peripheral blood cells. Labeling of cancer cells was demonstrated via flow cytometry in purified systems, as well as on an immobilized-cell platform similar to what is currently used in some CTC detection platforms. This work is the first step in the development of a function-based CTC labeling strategy to determine whether CTCs are procoagulant, and whether CTC enumeration and procoagulant characterization strategies are clinically useful in predicting thrombosis in patients with cancer.

## Conflict of Interest Statement

The authors declare that the research was conducted in the absence of any commercial or financial relationships that could be construed as a potential conflict of interest.
